# Supplementary material for: Flexible Fiber Membrane Based on Carbon Nanotube and Polyurethane with High Thermal Conductivity
Source: Nanomaterials (Basel). 2021 Sep 26;11(10):2504. doi: 10.3390/nano11102504 (PMC8538300; doi:10.3390/nano11102504)
Supplement: Supplementary file 1 [file nanomaterials-11-02504-s001.zip › nanomaterials-1382738-supplementary.pdf]

SUPPORTING INFORMATION

Flexible Fiber Membrane Based on Carbon Nanotube and Polyurethane with High Thermal Conductivity

Yuanzhou Chen [1,†], Junlin Chen [1,†], Yingming Zhang [1], Ziyue Hu [1], Weijian Wu [1], Xiang Chen [1,*] and Zhifeng Hao [1,2,*]

[1] School of Chemical Engineering and Light Industry, Guangdong University of Technology, Guangzhou 510006, China; m15521162019@163.com (Y.C.); 2112106060@mail2.gdut.edu.cn (J.C.); zhuos510@163.com (Y.Z.); huzydmn@163.com (Z.H.); w13247361677@163.com (W.W.)

[2] Guangdong Provincial Key Laboratory of Plant Resources Biorefinery, Guangdong University of Technology, Guangzhou 510006, China

* Correspondence: chenxiang@gdut.edu.cn (X.C.); haozf@gdut.edu.cn (Z.H.)

† These authors contributed equally to this work.

Fourier transform infrared (FTIR) spectra measurements were performed with a resolution of 4 cm^{-1} and a range from 500 to 4000 cm^{-1} via KBr pressed disc technique.

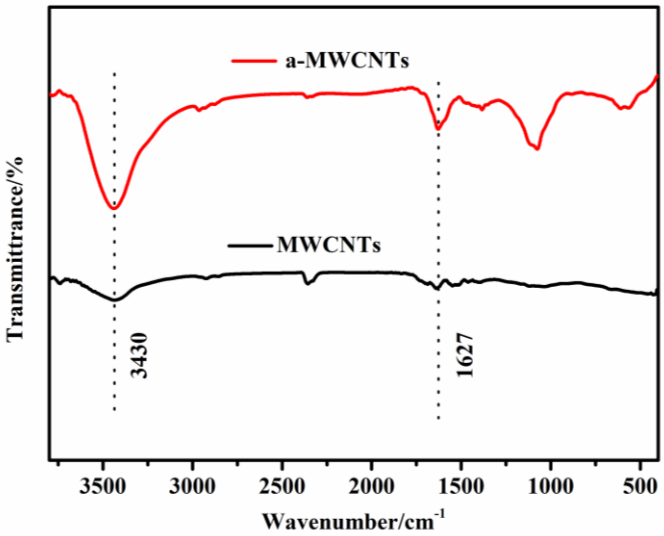

Figure S1 FTIR spectra of MWCNTs and a-MWCNTs.

Table S1 A comparison of the enhancement of thermal conductivity forCNTs-

based film-like composites.

Filler loading	Polymeric matrix	λ (W/mK)	Enhancement (%)	Ref.
2 wt% MWCNTs	PAN	4.825	234	[28]
35.3 wt% CNTs	NFC	19.3	1135	[36]
15 wt% CNTs	PVDF	1.12	747	[37]
4 wt% MWCNTs	PI	0.63	461	[38]
10 wt% MWCNTs	PA6	1.159	232	[39]
5 wt% CNTs	EP	1.0	441	[19]
10 wt% MWCNTs	TPU	3.60	1800	Our work
